# Inadequate Status of Multiple B Vitamins is Common Among Women in Oromia Region, Ethiopia

**DOI:** 10.1016/j.cdnut.2025.107578

**Published:** 2025-10-17

**Authors:** Christine M McDonald, Masresha Tessema, Isaac Agbemafle, Meseret Woldeyohannes, Mengistu Fereja, Kerry S Jones, Charles D Arnold, Biniyam Tesfaye, Mandana Arabi, Homero Martinez, Kenneth H Brown

**Affiliations:** 1 Department of Pediatrics, University of California San Francisco, Oakland, CA, United States; 2 Department of Nutrition and Institute for Global Nutrition, University of California, Davis, CA, United States; 3 Nutrition, Environmental Health and Non-communicable Diseases Research Directorate, Ethiopian Public Health Institute, Addis Ababa, Ethiopia; 4 Department of Nutrition, University of Rhode Island, Kingston, RI, United States; 5 Fred N. Binka School of Public Health, University of Health, and Allied Sciences, PMB 31, Ho, Ghana; 6 Nutritional Biomarker Laboratory, MRC Epidemiology Unit, University of Cambridge, Cambridge, United Kingdom; 7 Research & Development Unit, Nutrition International, Ottawa, ON, Canada

**Keywords:** micronutrients, folate, vitamin B12, thiamine, riboflavin, undernutrition, women’s nutrition, Ethiopia

## Abstract

The true burden of micronutrient deficiencies among women of reproductive age (WRA) in many resource-limited settings is likely underestimated, as data on several “neglected micronutrients” remain sparse. We used data from a cross-sectional survey of 100 nonpregnant WRA in the region of Oromia, Ethiopia to describe the prevalence of various micronutrient deficiencies and other indicators of nutritional status. The prevalences of anemia (hemoglobin < 13.4 g/dL, adjusted for altitude) and iron deficiency (inflammation-adjusted serum ferritin < 15 ug/L) were 7.1% and 4.0%, respectively. However, 100.0% of females were deficient in riboflavin [erythrocyte glutathione reductase activation coefficient (EGRac) > 1.4], and the prevalences of thiamine insufficiency [erythrocyte transketolase activity coefficient (ETKac) 1.15–1.25], folate insufficiency [red blood cell (RBC) folate < 748 nmol/L], and vitamin B12 insufficiency (serum B12 < 221 pmol/L) were 38.8%, 96.0%, and 52.6%, respectively. Future surveys should include a more comprehensive assessment of B vitamins to corroborate these findings and inform the design of appropriate interventions to improve status.

## Introduction

Micronutrient deficiencies are associated with several adverse outcomes in women and their offspring, including increased morbidity, impaired cognition and work productivity, and an increased risk of preterm birth, intrauterine growth restriction, and neural tube defects (NTDs). Globally, an estimated 69% of nonpregnant women of reproductive age (NPWRA) experience at least one of three essential micronutrients (iron, zinc, and folate) [1]. However, these figures likely underestimate the true burden of micronutrient deficiencies, because they do not capture deficiencies in “neglected micronutrients”, such as thiamine, riboflavin, vitamin B12, and others that are seldom measured in dietary assessments, surveys of micronutrient status, or public health surveillance systems. In a recent narrative review, Brown et al. [2] called for greater attention to neglected micronutrients to generate better data on the prevalence of deficiency and to design appropriate strategies to control deficiency.

In Ethiopia, the 2021–2022 Food and Nutrition Strategy Baseline Survey documented that the national prevalence of folate insufficiency [defined as a red blood cell (RBC) folate concentration <748 nmol/L measured using the United States Centers for Disease Control and Prevention microbiological assay] was 58.5% [3]. The country also exhibits a national NTD prevalence of 60–63 per 10,000 live births, which is more than three times the global prevalence [[4], [5], [6]]. However, data on other neglected micronutrients are sparse. To help fill this evidence gap, we utilized data from a cross-sectional survey of 100 NPWRA in the region of Oromia, Ethiopia to describe the prevalence of various micronutrient deficiencies and other indicators of nutritional status [7]. We also examined measures of association between biomarkers of micronutrient status in this population.

## Methods

### Study design and setting

This cross-sectional survey was conducted in the Gimbichu woreda of the Oromia region of Ethiopia from June to August 2023. The primary objectives of this survey were to assess the micronutrient status and estimate daily discretionary salt intake of NPWRA in the study area to inform the design of a subsequent randomized controlled trial of salt fortified with iodine and folic acid. The study setting has been described previously [7].

### Screening and enrollment procedures

A census of all households in the 2 semiurban kebeles was conducted in collaboration with the district health office, and we extracted a list of households with WRA from an existing list of households in the rural kebeles that was provided by health posts. We excluded households that did not have a WRA and/or did not plan to stay in the study area for at least one month. Potentially eligible WRA were listed sequentially by kebele, and we used a random number generator to select individual WRA from each kebele using a probability proportional to size sampling approach.

Our research team visited selected females at home to explain the study objectives and share information about the study procedures. Verbal consent was obtained for the screening process, and a questionnaire was administered to confirm the following eligibility criteria: *1)* woman’s reported age between 18–49 y; *2*) willingness to use the salt provided by the research team for a household salt disappearance study; *3*) not currently pregnant (based on self-report); *4*) not experiencing a self-reported acute or chronic disease that might affect dietary intake or folate status; *5*) not currently using medications that may affect folate metabolism; and *6*) not under medical prescription to restrict salt intake. Because the survey was conducted to inform the design of a subsequent randomized trial of salt fortified with folic acid and iodine, the inclusion criteria were similar for both activities.

### Data collection

Eligible females were asked to discuss potential participation with their family members before the research team’s subsequent household visit. At that visit, written informed consent was obtained, a questionnaire was administered to collect background characteristics, and an appointment was scheduled for a subsequent visit that occurred at a central location in the community where blood was drawn, anthropometric measurements were performed, and blood pressure was assessed.

A fasting blood sample (∼16 mL whole blood) was collected aseptically from the antecubital vein by experienced phlebotomists using evacuated tube blood collection tubes (BD). Six mL of blood was collected into a potassium EDTA tube for hemolysates for RBC folate determination; and whole blood for a complete blood count, blood smear for morphologic examination and preparation of washed RBCs for determination of the erythrocyte glutathione reductase activity coefficient (EGRac) and erythrocyte transketolase activity coefficient (ETKac). The remaining blood was collected into serum separator tubes and allowed to clot for 30 min in a cooler and protected from light. The blood specimens for serum samples were centrifuged at 3000 *g* for 10 min at the field site using a portable centrifuge, transferred into multiple cryotubes and temporarily stored at −20°C, then transported to the central laboratory at the Ethiopian Public Health Institute (EPHI) for long-term storage at −80°C.

Trained anthropometrists measured weight, height, midupper arm, waist, and hip circumferences of all women using standard procedures [8]. Following a rest period of ≥15 min, blood pressure was measured twice ≥3 min apart, using an OMRON silver automated blood pressure monitor (OMRON model BP5250). High blood pressure was defined as systolic pressure >140 mm Hg or diastolic pressure >90 mm Hg [9].

### Laboratory analysis

A complete blood count was performed using an automated analyzer (Sysmex Model XQ-320) and Sysmex-Eightcheck-3WP standards at the field laboratory. RBC folate and serum folate concentrations were measured at EPHI using a microbiological assay, as adapted by the United States CDC, and acceptable results were confirmed through participation in the United States CDC’s VITAL-external quality assurance and method performance verification programs [10]. The microbiological assay was performed in quadruplicate using a chloramphenicol-resistant strain of *Lactobacillus rhamnosus* and a 5-methyl-tetrahydrofolate calibrator. Serum homocysteine, vitamin B12, holotranscobalamin, thyroglobulin, glucose and insulin concentrations were measured at EPHI on a COBAS 6000 Analyzer with the following respective assay controls: homocysteine control kit Level 1/2, PreciControl Varia Level 1/2, PreciControl Active B12 Level 1/2, PreciControl Universal Level 1/2, PreciControl Clin Chem Level 1/2, and PreciControl Universal Level 1/2. Ferritin, soluble transferrin receptor (sTfR), C-reactive protein, and α-1-acid glycoprotein were analyzed using a multiplex immunoassay at the VitMin laboratory in Willstaett, Germany with controls from the United States CDC VITAL-external quality assurance [11]. EGRac and ETKac were measured at the University of Cambridge. These assays measure the activities of riboflavin and thiamine dependent enzymes by spectrophotometer in 96-well plate format [12,13]. Pooled blood from a single donor spiked with flavin adenine dinucleotide (FAD) and NAD, respectively, was used as a standard. The coefficient of variation for the low and high concentrations of assay controls were <10% in all cases except for serum B12, which was 12.5% and 12.9%, respectively.

We defined deficiency/insufficiency of various micronutrients using the following cutoffs: low serum folate concentration [14,15]: serum folate < 10 nmol/L; folate deficiency: RBC folate < 317 nmol/L [16]; folate insufficiency: RBC folate < 748 nmol/L; vitamin B12 deficiency: serum B12 < 150 pmol/L; vitamin B12 insufficiency: serum B12 150–220 pmol/L; low holoTC: holoTC < 40 pmol/L; elevated methylmalonic acid (MMA): >376 nmol/L; elevated homocysteine: > 15 umol/L; riboflavin deficiency [17]: EGRac > 1.4; high risk of thiamine deficiency [12]: ETKac > 1.25; thiamine insufficiency: ETKac 1.15–1.25; iron deficiency [18,19]: inflammation-adjusted ferritin < 15 ug/L, inflammation-adjusted sTfR > 5.3 mg/L; anemia [20]: hemoglobin < 13.4 g/dL (adjusted for altitude); iron deficiency anemia: inflammation-adjusted ferritin < 15 ug/L and hemoglobin < 13.4 g/dL (adjusted for altitude); and high glucose: fasting blood glucose > 125 mg/dL [21].

### Statistical analysis

Our sample size of 100 NPWRA was based on logistical and cost constraints. Assuming the 2016 national folate insufficiency prevalence of 74% [22], we estimated that this sample size would enable us to detect a 95% confidence interval of 64.3%–82.3%.

We used descriptive statistics to summarize sociodemographic characteristics and various indicators of nutritional status. Frequencies were reported for categorical variables, means and SDs were reported for normally distributed continuous variables, and medians (interquartile range) were reported for nonnormally distributed continuous variables. To examine the association between biomarkers of micronutrient status, scatterplots were generated and Spearman correlation analyses were conducted. A complete-case approach was taken due to low rates of missing data. SAS version 9.4 and/or Stata version 16.1 were used for all analyses.

### Ethical statement

The study protocol was approved by the Institutional Review Boards of the Ethiopian Public Health Institute on June 5, 2023, and the University of California, Davis on March 21, 2023. The study was registered at clinicaltrials.gov (NCT1834465-1).

## Results

Mean participant age was 31 y and only 35% had completed primary school or higher (data not shown). Three-quarters of females were married or living with their partner, and 56% did not work out outside the home. Mean ± SD household size was 4.9 ± 1.8 people, and 44% of households experienced some degree of food insecurity. Table 1 summarizes the nutritional status of the study participants. Mean ± SD BMI was 22 ± 3.4 kg/m^2^. The burden of inflammation was relatively low: the proportion of females with C-reactive protein > 5 mg/L and α-1-acid glycoprotein > 1 g/L was 6.1% and 12.1%, respectively. The proportion of females with a systolic blood pressure > 140 mmHg and diastolic blood pressure > 90 mmHg was 4.0% and 18.2%, respectively. The vast majority of females had normal blood glucose and insulin parameters.

**TABLE 1:** Nutritional status of study participants (*n* = 100).

	Mean ± SD, Median (IQR) or *N* (%)
Height, cm	159 ± 5.3
Weight, kg	56 ± 9.5
BMI, kg/m^2^	22 ± 3.4
<18.5	12 (12)
18.5–24.9	66 (66)
≥25	22 (22)
Inflammation	
C-reactive protein, mg/L	0.6 (0.3, 1.6)
C-reactive protein > 5 mg/L	6 (6.1)
α-1-acid glycoprotein, g/L	0.7 (0.6, 0.9)
α-1-acid glycoprotein > 1 g/L	12 (12.1)
Elevated C-reactive protein or α-1-acid glycoprotein	15 (15.2)
Blood pressure	
Systolic blood pressure, mmHg	115.2 ± 14.4
Systolic blood pressure > 140 mmHg	4 (4.0)
Diastolic blood pressure, mmHg	82.7 ± 9.7
Diastolic blood pressure > 90 mmHg	18 (18.2)
Blood glucose control	
Glucose, mg/dL	90 ± 8
Glucose > 125 mg/dL	0 (0.0)
Insulin (uIU/mL)	8.7 (6.6, 12)
Insulin > 25 uIU/mL	1 (1.0)
HOMA, pancreatic β-cell function (%)	126 (85, 159)
HOMA-IR, insulin resistance (R value)	1.9 (1.4, 2.7)
Hemoglobin, g/dL	14.8 ± 1.1
Hemoglobin < 13.4 g/dL (cutoff adjusted for altitude)	7 (7.1)
Iron deficiency anemia	2 (2.0)
Macrocytosis (Mean corpuscular volume > 100 fL)	1 (1.0)
Microcytosis (Mean corpuscular volume < 80 fL)	2 (2.0)
Iron	
Ferritin (unadjusted), μg/L	79.2 (55.2, 123.8)
Ferritin (inflammation-adjusted)	64.9 (41.2, 87.3)
Ferritin (inflammation-adjusted) < 15 μg/L	4 (4.0)
Soluble transferrin receptor (unadjusted), mg/L	4.8 (4.2, 5.6)
Soluble transferrin receptor (inflammation-adjusted), mg/L	4.4 (3.6, 4.9)
Soluble transferrin receptor (inflammation-adjusted) > 8.3 mg/L	18 (18.2)
Body iron stores, mg/kg^8^	8.2 ± 3.2
Body iron stores < 0 mg/kg	2 (2.0)
Vitamin B12	
Serum B12, pmol/L	215.5 (175.5, 287.7)
Serum B12 < 150 pmol/L	16 (16.2)
Serum B12 150–220 pmol/L	36 (36.4)
Serum B12 > 221 pmol/L	47 (47.5)
Holo transcobalamin, pmol/L	48.4 (39.7, 53.3)
Holo transcobalamin < 40 pmol/L	27 (27.3)
Homocysteine, μmol/L	8.8 (7.1, 11)
Homocysteine > 15 μmol/L	8 (8.1)
Methylmalonic acid, nmol/L	373.7 (184.1, 636.9)
Methylmalonic acid > 376 nmol/L	49 (50)
Composite B12 indicator	−0.3 (−0.7, 0.1)
Composite B12 indicator < −0.5	35 (35.7)
Folate	
Serum folate, nmol/L	14.8 (11.9, 18.4)
Serum folate < 10 nmol/L	15 (15.2)
Red blood cell folate, nmol/L	466.4 (386.5, 551.5)
Red blood cell folate < 748 nmol/L	95 (96.0)
Red blood cell folate < 317 nmol/L	12 (12.1)
Riboflavin (n = 80)	
Erythrocyte glutathione reductase activation coefficient	2.3 ± 0.3
Erythrocyte glutathione reductase activation coefficient < 1.2	0 (0.0)
Erythrocyte glutathione reductase activation coefficient 1.2–1.4	0 (0.0)
Erythrocyte glutathione reductase activation coefficient > 1.4	80 (100)
Thiamine (n = 80)	
Erythrocyte transketolase activity coefficient	1.1 (1.1, 1.2)
Erythrocyte transketolase activity coefficient < 1.15	49 (61.3)
Erythrocyte transketolase activity coefficient 1.15–1.25	31 (38.8)
Erythrocyte transketolase activity coefficient > 1.25	0 (0.0)
Iodine	
Thyroglobulin, ng/mL	15.4 (9.4, 22.7)
Urinary iodine, μg/L	253.7 (193.3, 334.8)

The prevalences of anemia, iron deficiency (inflammation-adjusted ferritin < 15 ug/L) and iron deficiency anemia were 7.1%, 4%, and 2%, respectively. At 1% and 2%, the respective prevalences of macrocytosis and microcytosis were low. However, more than half of study participants were classified as vitamin B12 insufficient or deficient according to a serum B12 < 221 pmol/L. Likewise, 50% of females had elevated MMA. More than one-quarter of females had a holoTC < 40 pmol/L, and 8.1% of females had hyperhomocysteinemia. More than one-third of females had a low composite B12 (<−0.5), an indicator which incorporates all 4 biomarkers of vitamin B12 status [23]. At 96.0%, folate insufficiency (RBC folate < 748 nmol/L) was almost universal. The prevalence of folate deficiency (RBC folate < 317 nmol/L) was 12.1%. All women with available samples were deficient in riboflavin (EGRac > 1.4) and 38.8% had thiamine insufficiency (ETKac 1.15-1.25).

Table 2 summarizes the correlation among biomarkers of micronutrient status. As expected, ferritin was inversely associated with sTfR (*r* = −0.32, *P* = 0.004). STfR (*r* = −0.26, *P* = 0.02) was associated with MCV, but ferritin was not. There was a positive, but modest association between serum folate and RBC folate (*r* = 0.27, *P* = 0.02). Likewise, there was a moderate correlation among all 4 biomarkers of vitamin B12 status and the composite B12 indicator. Among these, the weakest correlation was observed between holoTC and homocysteine (*r* = −0.22, *P* = 0.05) and the strongest was observed between MMA and the composite B12 indicator (*r* = −0.89, *P* < 0.001). The correlations between MCV and MMA (*r* = 0.28, *P* = 0.006) and the composite B12 indicator (*r*= −0.30, *P* < 0.001) were both highly significant, whereas the association between serum B12 and MCV was marginally significant (*r* = −0.22, *P* = 0.05). EGRac and ETKac were not associated with each other. There was also a positive correlation between EGRac and MCV (*r* = 0.26, *P* = 0.02). EGRac was negatively associated with serum B12 (*r* = −0.25, *P* = 0.03), which was not surprising as higher EGRac values reflect poorer riboflavin status and both nutrients share some food sources. Otherwise, neither EGRac nor ETKac were associated with any other biomarkers of folate or vitamin B12 status.

**TABLE 2:** Correlation among biomarkers of folate and vitamin B12 status.

	Ferritin^1^	sTfR^1^	MCV	Serum folate	RBC folate	Serum B12	HoloTC	MMA	Homocysteine	Composite B12 indicator	ETKac	EGRac
Ferritin^1^	1											
sTfR^1^	*r* = −0.32 *P* = 0.004	1										
MCV	*r* = 0.15 *P* = 0.14	*r* = −0.26 *P* = 0.02	1									
Serum folate	*r* = 0.20 P = 0.08	*r* = −0.17 *P* = 0.14	*r* = −0.02 *P* = 0.85	1								
RBC folate	*r* = 0.08 *P* = 0.47	*r* = 0.02 *P* = 0.89	*r* = −0.10 *P* = 0.31	*r* = 0.27 *P* = 0.01	1							
Serum B12	*r* = −0.11 *P* = 0.32	*r* = 0.13 *P* = 0.25	*r* = −0.22 *P* = 0.05	*r* = 0.05 *P* = 0.64	*r* = 0.33 *P* = 0.003	1						
HoloTC	*r* = −0.01 *P* = 0.97	*r* = 0.09 *P* = 0.41	*r* = −0.20 *P* = 0.07	*r* = 0.03 *P* = 0.08	*r* = 0.26 *P* = 0.02	*r* = 0.20 *P* = 0.07	1					
MMA	*r* = 0.05 *P* = 0.64	*r* = 0.02 *P* = 0.85	*r* = 0.28 *P* = 0.006	*r* = 0.04 *P* = 0.75	*r* = −0.39 *P* < 0.001	*r* = −0.38 *P* < 0.001	*r* = 0.41 *P* < 0.001	1				
Homocysteine	*r* = 0.03 *P* = 0.78	*r* = 0.17 p = 0.13	*r* = 0.18 *P***=** 0.11	*r* = −0.21 p = 0.07	*r* = −0.35 *P* = 0.003	*r* = −0.23 *P* = 0.04	*r* = −0.22 *P* = 0.05	*r* = 0.09 *P* = 0.42	1			
Composite B12 indicator	*r* = −0.05 *P* = 0.65	*r* = −0.01 p = 0.98	*r* = −0.30 *P* < 0.001	*r* = 0.03 p = 0.76	*r* = 0.48 *P* < 0.001	*r* = 0.62 *P* < 0.001	*r* = 0.56 *P* < 0.001	*r* = −0.89 *P* < 0.001	*r* = −0.39 *P* < 0.001	1		
ETKac	*r* = 0.18 *P* = 0.12	*r* = −0.16 *P* = 0.15	*r* = 0.05 *P* = 0.62	*r* = −0.06 *P* = 0.62	*r* = 0.03 *P* = 0.78	*r* = −0.12 v = 0.31	*r* = 0.01 *P* = 0.92	*r* = −0.04 *P* = 0.72	*r* = −0.02 *P* = 0.86	*r* = −0.03 *P* = 0.86	1	
EGRac	*r* = −0.06 *P* = 0.58	*r* = 0.04 *P* = 0.73	*r* = 0.26 *P* = 0.02	*r* = −0.11 *P* = 0.35	*r* = −0.15 *P* = 0.18	*r* = −0.25 *P* = 0.03	*r* = 0.08 *P* = 0.48	*r* = 0.13 *P* = 0.27	*r* = 0.20 *P* = 0.07	*r* = −0.20 *P* = 0.07	*r* = 0.12 *P* = 0.28	1

Abbreviations: sTfR, soluble transferrin receptor; RBC folate, red blood cell folate; HoloTC, holo transcobalamin; MMA, methylmalonic acid; ETKac, erythrocyte transketolase activity coefficient; EGRac, erythrocyte glutathione reductase activity coefficient.

1 Adjusted for inflammation using the BRINDA regression correction approach.

## Discussion

In this study involving 100 NPWRA in Oromia region, Ethiopia, we observed a high prevalence of insufficiency in multiple B vitamins. All women were deficient in riboflavin, 96.0% had folate insufficiency, 38.8% were insufficient in thiamine, and 52.6% had vitamin B12 insufficiency. These alarmingly high prevalence estimates of insufficiency underscore the urgent need for more extensive data at the national and regional level to inform the design of effective nutrition interventions to improve intake and status of these B vitamins.

Although national-level data on folate and vitamin B12 status have been reported as part of the Ethiopian National Micronutrient Survey [22], to our knowledge, our study is the first to report population-based data on riboflavin and thiamine status of NPWRA in Ethiopia. These B vitamins can be categorized as “neglected micronutrients” given the dearth of existing prevalence data and their typical exclusion from large-scale, public health nutrition programs [2]. Thiamine plays an essential role in energy metabolism and severe thiamine deficiency can result in beriberi, a clinical syndrome characterized by anorexia, congestive heart failure and/or neurologic dysfunction [24]. Although we did not identify females who were deficient in thiamine (ETKac >1.25), 38% had an ETKac between 1.15 and 1.25, which indicates thiamine insufficiency. Riboflavin functions as 2 coenzymes, FAD and flavin mononucleotide, which are also involved in energy metabolism and cellular antioxidant function [17]. Severe riboflavin deficiency presents clinically as cheilosis, glossitis, angular stomatitis, seborrheic dermatitis, and severe anemia with erythroid hypoplasia. The prevalences of both microcytic anemia and macrocytic anemia were very low in our study population and EGRac was not significantly associated with ferritin or sTfR. However, we observed a negative association between EGRac and serum B12 and a positive association between EGRac and MCV, which is relevant as riboflavin is metabolically related to vitamin B12 (and other B vitamins) [25]. FAD is required as a cofactor for the formation of 5-methyltetrahydrofolate, which is in turn, a methyl donor in the remethylation of homocysteine to methionine, a reaction that is catalyzed by methione synthase using vitamin B12 as a cofactor. Emerging evidence also suggests that riboflavin, a cofactor of the MTHFR gene, may have a genotype-specific effect on blood pressure [26]. In our study population, EGRac was not correlated with systolic (*r* = 0.06, *P* = 0.60) or diastolic blood pressure (*r* = −0.02, *P* = 0.89); however, only 2% of participants were homozygous for the 677 T/T polymorphism in the MTHFR gene.

Dairy foods are the main dietary source of riboflavin. Although not reported here, our study involved the collection of one-day weighed food records in all 100 study participants, with repeat assessments in a subset of 40 participants. Micronutrient analysis of the dietary data was not conducted; however, the weighed food records indicate that only 4% of participants consumed any milk, yogurt, or cheese on the day of their dietary assessment, which is consistent with the high levels of functional riboflavin deficiency observed in the biochemical data. The combination of high-quality dietary intake data and biochemical data on micronutrient status is essential to understand the etiology of insufficiency and/or deficiency and to design effective programs for control.

The 96% prevalence of folate insufficiency observed in our study population is higher than the national average of 58.5% reported in the 2021–2022 Ethiopia Food and Nutrition Strategy Baseline Survey. In response to the concerningly high prevalence of folate insufficiency and the related burden of NTDs, the Government of Ethiopia instituted mandatory fortification of wheat flour with folic acid, thiamine, riboflavin, niacin, vitamin B6, and vitamin B12 in 2022. However, because the consumption of centrally processed wheat flour is limited among rural populations with modest socioeconomic means, it is unlikely that this approach will be very effective in improving status of folate and other B vitamins among populations at greatest risk of deficiency. Salt is being explored as an alternative fortification vehicle for folic acid, because it is widely consumed across socioeconomic strata and geographies, and folic acid can be combined with the potassium iodate spray that is currently used to iodize salt [27]. Although additional B vitamins are not currently being considered for co-fortification of salt in Ethiopia, future research on this topic is warranted.

Our study has a number of strengths and limitations. We used recommended laboratory methods to assess a comprehensive suite of micronutrient biomarkers. Folate status was assessed using both serum and RBC folate, and the United States CDC microbiological assay was employed for analysis. Vitamin B12 status was assessed using four recommended biomarkers. Our assessment of thiamine and riboflavin status is also unique in this study setting. Unfortunately, resource and logistical constraints restricted the sample size and geographic coverage of the current study, which limits the generalizability of our findings. In addition, sample processing issues further reduced the number of participants for whom thiamine and riboflavin status could be analyzed.

In conclusion, our study revealed a high prevalence of insufficiency in multiple B vitamins among WRA in Oromia region, Ethiopia. The inclusion of riboflavin and thiamine status assessment should be considered in future surveys to corroborate these findings and to inform the design of appropriate interventions to improve status.

## Author contributions

CMM designed the research, reviewed statistical analyses, and wrote the manuscript; MT designed the research and supervised project implementation; IA conducted the research, and supervised project implementation; MW concducted the research, supervised project implementation, provided essential materials, and analyzed the data; MF conducted research and supervised project implementation; KSJ conducted laboratory analyses; CDA performed statistical analyses; BT contributed to research design; MA supervised project implementation; HM supervised project implementation; KHB desgined the research and supervised project implementation. All authors read and approved the final manuscript and share reponsibility for its content.

## Funding

Financial support for the study was provided by Nutrition International through a grant from the Gates Foundation.

## Conflict of interest

Homero Martinez and Mandana ARabi are employees of Nutrition International; they reviewed the study design, participated in meetings of the core research team to assess progress, and critically reviewed the draft manuscript. The corresponding author was responsible for the decision to submit the manuscript and there were no restrictions on publication.
